# Alternative 5‐Azacitidine 5‐Day 100 mg/m^2^ Dosage Shows Non‐Inferiority to Classical Schedule for Myelodysplastic Neoplasm (MDS) and Chronic Myelomonocytic Leukaemia (CMML) Treatment

**DOI:** 10.1002/jha2.70185

**Published:** 2025-11-26

**Authors:** Gerasimos Tsilimidos, Filipe Martins, Mariangela Costanza, Mathilde Gavillet, Sabine Blum

**Affiliations:** ^1^ Hematology Service and Central Laboratory of Hematology Lausanne University Hospital and University of Lausanne Lausanne Switzerland; ^2^ Clinics of Medical Oncology Cantonal Hospital of Fribourg (HFR) Fribourg Switzerland; ^3^ School of Life Sciences, École Polytechnique Fédérale de Lausanne (EPFL) Lausanne Switzerland

**Keywords:** azacitidine, CMML, MDS

## Abstract

**Introduction:**

5‐Azacitidine (AZA) is a major treatment option for myelodysplastic neoplasms (MDS) and myelodysplastic/myeloproliferative neoplasms (MDS/MPN). Here we evaluate the efficacy and toxicity of an alternative AZA regimen (100 mg/m^2^/day for 5 days/28 days) in 68 patients (51 MDS, 17 MDS/MPN) treated between 2008 and 2018.

**Results:**

Median patient age was 66 years, with most patients (98%) having intermediate or high‐risk disease. Overall response rate (ORR) was 62% with 22% complete responses (CR). Median OS and median PFS were 22.5 and 18.2 months, respectively. Inferior response rates were calculated in therapy‐related MDS (t‐MDS) and MDS with excess blast II, with t‐MDS having also statistically worse OS and PFS. MDS/MPN patients showed 73.6% ORR with 31.5% CR. Transfusion independence (TI) for red blood cells (RBC) was achieved in 45.9% of transfusion‐dependent patients and in 30% for platelets. CR patients showed longer mOS and mPFS (70.6 and 64.7 months, respectively). Longer mOS was also correlated with allogeneic transplantation (48.8 vs. 16.9 months, *p* = 0.01) and RBC TI (25.4 vs. 13.3 months, *p* = 0.01). Grade 3/4 cytopenias occurred in 41.1% (neutropenia in 33.8%), and treatment‐related mortality was 7.4%.

**Conclusion:**

This study demonstrates that this alternative AZA regimen has comparable efficacy and safety to the standard regimen, compared with historical data.

**Trial Registration:**

The authors have confirmed clinical trial registration is not needed for this submission.

## 1 Introduction

5‐Azacitidine (AZA) is the first hypomethylating agent, synthesized in 1964 as a nucleoside analogue [1]. Its cytotoxic properties at higher doses (> 2000 mg/m^2^) lead to DNA, RNA and protein synthesis inhibition and interference with de novo thymidylate synthesis when deaminated into 5‐azauridine [1, 2]. However, at lower doses, its most prominent effect is the rapid loss of DNMT (DNA cytosine‐C5 methyltransferase), mainly DNMT1, as the enzyme becomes irreversibly bound to 5‐azacytosine residues and degraded, resulting in a rapid loss of DNA methylation at each cell division, leading to toxic and mutagenic effects [3, 4].

Initially used for its cytotoxic properties in acute myeloid leukaemia (AML) treatment [5], AZA was then studied in the treatment of myelodysplastic neoplasia (MDS) during the 1990s, as an IV or subcutaneous administration for 7 days at a dose of 75 mg/m^2^ (7‐0‐0) every 4 weeks [6]. In 2009, the phase 3 AZA‐001 study validated the superiority of AZA monotherapy versus best supportive care (BSC) or conventional care regimens (CCRs) with a significantly better overall survival (OS) (24.5 vs. 15 months) for AZA [7]. It has since been rapidly approved by the Food and Drug Administration and European Medicines Agency as a monotherapy for the treatment of MDS with the aforementioned administration schedule.

Nowadays, AZA is used as a first‐line treatment for high‐risk MDS (HR‐MDS), but also represents a valid therapeutic option for chronic myelomonocytic leukaemia (CMML) with growing but conflicting evidence [8, 9].

Since the approval of the standard dose, many, mostly retrospective, studies have investigated different AZA regimens in order to compare their toxicity and efficacy profiles. The most commonly used alternative schedules usually use either the 75 mg/m^2^ dosage over 5 days (5‐0‐0) instead of 7 days, or 7 days but with a weekend interruption (5‐2‐2). The results from all of these different studies are inconclusive concerning efficacy and survival, with only some real‐life retrospective studies comparing these three schedules and reporting no OS difference [10].

Higher AZA doses of 100 mg/m^2^ have mostly been tested for AML, but data are scarce and unclear in the setting of MDS. The higher dose of 100 mg/m^2^ is recommended in cases of insufficient response after two cycles.

In this single‐centre retrospective study, we examined the efficacy and toxicity of the alternative AZA schedule of 100 mg/m^2^ for 5 days in a 10‐year patient cohort.

## 2 Methods

### 2.1 Study Population

This study is an observational, retrospective single‐centre study. We included all adult (> 18 years old) patients who received AZA monotherapy as a first or subsequent line of treatment for MDS or CMML between 1 January 2008 and 31 December 2018. Diagnosis was initially established according to either the 2008 or the 2016 WHO criteria [11, 12] and then homogenized according to the 2016 criteria. All patients received at least one dose of AZA. Only patients treated with the 5‐day 100 mg/m^2^ regimen were included. Follow‐up was pursued until December 2023. The last follow‐up was defined as the date of death or the last follow‐up. All patients were entirely or partially treated at Lausanne University Hospital (Centre Hospitalier Universitaire Vaudois [CHUV], Switzerland). This study was approved by the ethics committee of the Canton de Vaud under project number CER‐VD n.2018‐01391.

### 2.2 Objectives

The primary objective was overall response rate (ORR), defined as the sum of complete remission (CR) and partial remission (PR) according to IWG 2006 MDS response criteria [13]. Stable disease (SD) and progressive disease (PD) were also calculated according to the same criteria. Secondary objectives included OS, defined as the time between diagnosis and death or lost to follow‐up, and progression‐free survival, defined as the time between diagnosis and death, lost to follow‐up, or disease progression/relapse (also according to IWG 2006 MDS response criteria). Additional secondary objectives comprised the rate of red blood cell (RBC) and platelet (PLT) transfusion independence (TI) as defined by the 2018 revised IWG proposal [14] (i.e. > 16 weeks without transfusion), the rate of haematopoietic stem cell transplantation (HSCT), and the assessment of haematologic toxicities of Grades 3 and 4 as per CTCAE v6.0.

### 2.3 Data Collection

Patient file database (SOARIAN) was used to screen all patients treated between 1 January 2008 and 31 December 2018 with AZA and to collect clinical and biological data. In case of missing data, we referred to the hospital archives (Archimed software). Collaborating haematologists in private practice or from other Swiss hospitals were contacted to complete missing information for patients who continued treatment under their supervision.

### 2.4 Statistical Analysis

Statistics were performed using R (v 4.2.0; CRAN project, R Foundation for Statistical Computing, Vienna, Austria). We used the package ‘survival’ for analysis and the package ‘survminer’ [15] for the drawing of survival curves. We used the Kaplan–Meier estimator to report the survival probability and a multivariate Cox regression model to calculate hazard ratios (HRs). Median follow‐up was calculated using the reverse Kaplan–Meier method [16]. A log‐rank statistical test was applied to assess significance when comparing KM estimates. The significance of the covariates used in the multivariate Cox regression was assessed using Wald statistics as default in the coxph() function of the ‘survival’ package.

## 3 Results

### 3.1 Clinical Characteristics/Demographic Parameters of the Study Cohort

Overall, 68 patients were included with a median age of 66 years (range: 25–92) and 46 male patients (67.6%). Fifty‐one patients had MDS and 17 MDS/MPN. Fourteen had therapy‐related MDS (t‐MDS). Further subclassification according to WHO 2016 is shown in Table 1.

**TABLE 1:** Study population.

	Total	Women (22)	Men (46)
Median age (years)	66 (25–92)	65 (25–83)	66 (27–92)
Diagnosis (*N*)			
MDS	37	11	26
t‐MDS	14	6	8
CMML	15	5	10
MDS/MPN NOS	2	0	2
WHO 2016 classification (*N*)			
MDS‐MLD	4	1	3
MDS‐del5q	2	1	1
MDS‐EB1	13	3	10
MDS‐EB2	18	6	12
CMML‐0	2	0	2
CMML‐1	7	4	3
CMML‐2	6	1	5
MDS/MPN NOS	2	0	2
Risk scores (mean)			
	VL/L	INT	H/VH
IPSS‐R	1 (2%)	18 (36%)	31 (62%)
WPSS	2 (4%)	9 (18%)	39 (78%)
	L	INT 1/2	H
CPSS	2 (14%)	12 (86%)	0
CBC at diagnosis (median)			
HB (g/dL)	9.75 (5.1–14.3)	8.2 (5.1–14.3)	10.35 (5.4–14.3)
PLT (G/L)	75 (9–450)	64 (9–384)	79 (11–450)
ANC (G/L)	1.8 (0.14–21.3)	1.8 (0.19–21.3)	1.7 (0.14–15.7)
Blastes (%)	1 (0–15)	1.8 (0–15)	0 (0–14)
Bone marrow blastes (%)	8 (0–19)	9 (3–15)	8 (0–18)
TD RBC (*N*)	37	15	22
TD PLT (*N*)	27	10	17
Median no of cycles (*N*)	5 (1–134)	5 (1–95)	5 (1–134)
Line of treatment (*N*)			
1	47	16	31
2	15	5	10
≥ 3	12	3	9
Other treatments (*N*)			
IC	17	6	11
Valproic acid	3	0	3
HSCT/DLI	11	2	9
GO	1	0	1
Lenalidomide	5	2	3
DEC	2	0	2
5‐AZA	6	2	2
HSCT after 5‐AZA (*N*)	22	8	14

Abbreviations: ANC, absolute neutrophil count; AZA, azacitidine; CMML, chronic myelomonocytic leukaemia; CPSS, CMML specific prognostic scoring system; DEC, decitabine; DLI, donor lymphocyte infusion; EB, excess blasts; GO, gemtuzumab ozogamicin; HB, haemoglobin; HSCT, haematopoietic stem cell transplantation; IC, intensive chemotherapy; IPSS‐R, revised international prognostic system; MDS, myelodysplastic neoplasm; MLD, multilineage dysplasia; MPN, myeloproliferative neoplasm; NOS, non‐otherwise specified; PLT, platelets; RBC, red blood cells; TD, transfusion dependence; WPSS, WHO classification‐based prognostic scoring system.

IPSS‐R score was low (2.5 points) for 1 patient, intermediate for 18 patients and high or very high for 31 patients (only one patient's missing data did not allow for score calculation). CPSS score was low for 2 patients and intermediate 1/2 for 12 patients with also one patient having missing values. The median haemoglobin (Hb) level, PLT and neutrophil (ANC) counts at diagnosis were 9.75 g/dL, 75 G/L and 1.8 G/L, respectively. Median blast count was 1% (0–15) in peripheral blood and 8% (0–19) in the bone marrow at diagnosis.

Thirty‐seven patients were transfusion‐dependent (TD) for RBC (54.4%) and 27 for PLT (39.7%) at AZA initiation, while 23 (33.8%) were TD for both.

The median number of cycles was 5 (1–134), and 47 of 68 patients (69.1%) received AZA as a first‐line treatment.

### 3.2 AZA Treatment Outcome

Among the 68 included patients, 6 were re‐exposed to AZA at disease relapse after HSCT, accounting for 74 documented responses. The median follow‐up time was 98.6 months.

The ORR was 62% (46/74) for the whole cohort, with 22% of CR (16/74) and 41% of PR (30/74). Fifteen patients showed an SD (20%) and 13 had a PD (17%). A sub‐analysis found a lower ORR among MDS with excess blast II (EB II) and t‐MDS with 52.6% and 50%, respectively, as compared to all other MDS (ORR = 65%). CMML and MDS/MPN NOS had an ORR of 73.6% with 31.5% of CR. All responses are summarized in Table 2.

**TABLE 2:** Responses.

Responses, *N* (%)	
ORR	46/74 (62.1%)
CR	16/74 (21.6%)
PR	30/74 (40.5%)
SD	15/74 (20.2%)
PD	13/74 (17.5%)

By subgroup				
	MDS other	MDS‐EB II	t‐MDS	CMML, MDS/MPN NOS
ORR	13 (65%)	10 (52.6%)	8 (50%)	14 (73.6%)
CR	6 (30%)	4 (21%)	0	6 (31.5%)
PR	7 (35%)	6 (31.5%)	8 (50%)	8 (42.1%)
SD	2 (10%)	7 (36.8%)	5 (31.2%)	1 (5.2%)
PD	4 (20%)	2 (10.5%)	3 (18.7%)	4 (21%)

Abbreviations: CMML, chronic myelomonocytic leukaemia; CR, complete remission; EB, excess blast; MDS, myelodysplastic neoplasm; MPN, myeloproliferative neoplasm; NOS, non‐otherwise specified; ORR, overall response rate; PD, progressive disease; PR, partial remission; SD, stable disease; t‐MDS, therapy related MDS.

Median time to best response was 6.9 months for CR and 3.65 months for PR. The HRs calculated for patients without a CR were 2.86 (95% CI: [1.21–6.76]), 3.36 (95% CI: [1.3–8.5]) and 8.31 (95% CI: [3.2–21.7]) for PR, SD and PD, respectively.

Out of 37 RBC TD patients at AZA initiation, 17 achieved TI (45.9%) during treatment. The median time to RBC TI was 5.1 months (1.6–54). Out of 30 PLT TD patients, 9 achieved TI (30%) during treatment. The median time to PLT TI was 5.1 months (1.1–16.4).

Among the 68 treated patients, 26 received HSCT, 17 of them after AZA treatment (25%) and 9 (13.2%) after AZA as first‐line treatment.

### 3.3 Survival

**FIGURE 1:**
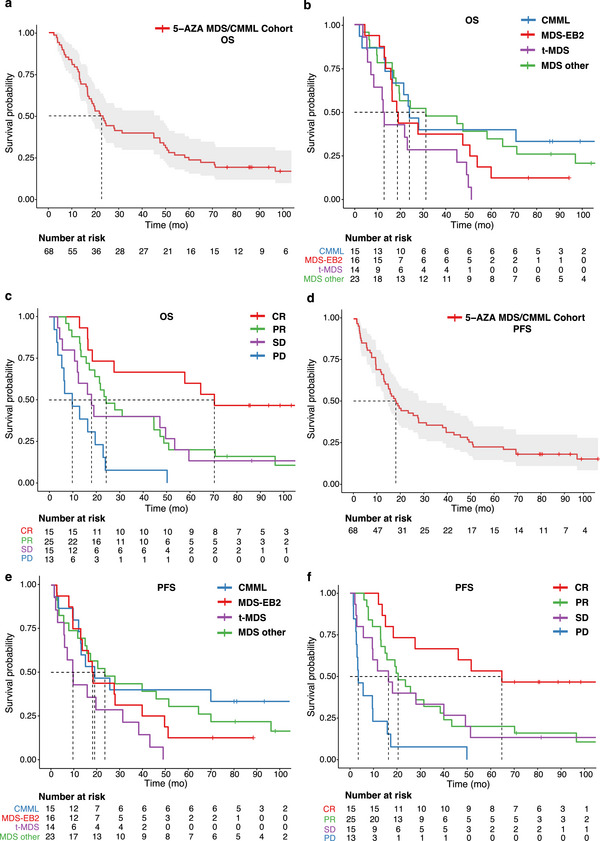
(a) Kaplan–Meier curve showing the overall survival (OS) of the 68 patients treated with 5‐AZA from 2007 to 2018 in our clinic. The mOS of the entire cohort was 22.5 months (95% CI: 17.2–47.3). (b) OS comparison between the groups diagnosed with CMML, MDS‐EB2, t‐MDS, and remaining MDS categories (i.e. MDS other) according to 2016 WHO classification. The mOS of CMML, MDS‐EB2, t‐MDS, and MDS other was 24.0 (95% CI: 16.6–NA), 18.8 (95% CI: 15.8–59.7), 12.8 (95% CI: 8.4–49.7) and 31.2 months (95% CI: 18.1–96.5), respectively. (c) OS comparison between the patient groups who achieved either a complete response (CR), partial response (PR), stable disease (SD) or progressive disease (PD) under 5‐AZA treatment according to IWG 2006 MDS response criteria. The mOS of CR, PR, SD and PD was 70.6 (95% CI: 27.7–NA), 24.4 (95% CI: 19.5–49.0), 18.1 (95% CI: 12.1–59.7) and 9.9 months (95% CI: 5.4–NA), respectively. (d) Kaplan–Meier curve showing the progression‐free survival (PFS) of the 68 patients treated with 5‐AZA from 2007 to 2018 in our clinic. The mPFS of the entire cohort was 18.2 months (95% CI: 13.7–31.5). (e) Progression‐free survival (PFS) comparison between the groups diagnosed with CMML, MDS‐EB2, t‐MDS and remaining MDS categories (i.e. MDS other) according to 2016 WHO classification. The mPFS of CMML, MDS‐EB2, t‐MDS and MDS other was 19.1 (95% CI: 13.0–NA), 18.3 (95% CI: 13.0–51.4), 9.6 (95% CI: 5.9–43.4) and 23.6 months (95% CI: 15.0–70.2), respectively. (f) PFS comparison between the patient groups who achieved either a complete response (CR), partial response (PR), stable disease (SD) or progressive disease (PD) under 5‐AZA treatment according to IWG 2006 MDS response criteria. The mPFS of CR, PR, SD and PD other was 64.7 (95% CI: 27.7–NA), 20.5 (95% CI: 15.0–40.0), 16.4 (95% CI: 9.5–51.4) and 3.5 months (95% CI: 2.7–NA), respectively.

Median OS (mOS) for entire cohort was 22.5 months (95% CI: 17.2–47.3, Figure 1a). Subgroup analysis showed a median OS for CMML, MDS EB‐II, t‐MDS and MDS others, at 24 (95% CI: 16.6–NA), 18.8 (95% CI: 15.8–59.7), 12.8 (95% CI: 8.4–49.7) and 31.2 (95% CI: 18.1–96.5) months, respectively (Figure 1b), with t‐MDS having a significantly worse prognosis compared to CMML (*p* = 0.01) and MDS others (*p* = .01) in pairwise comparisons. Patients achieving a CR had a significantly better prognosis, with mOS of 70.6 months, over patients with a PR (mOS 24.4 months), SD (mOS 18.1) or PD (mOS 9.9) (Figure 1c).

The median PFS (mPFS) for the whole cohort was 18.2 (95% CI: 13.7–31.5) months (Figure 1d). Similarly, MDS others (mPFS 23.6 months) and CMML (mPFS 19.1 months) had a significantly better PFS compared to t‐MDS (mPFS 9.6 months, *p* = 0.02/0.01) in contrast to MDS EB‐II (mPFS 18.3 months, *p* = 0.12) (Figure 1e). As with OS, CR patients reached an mPFS of 64.7 months which was significantly better as compared to either PR (mPFS 20.5 months), SD (mPFS 16.4 months) or PD (mPFS 3.5 months) patient groups (Figure 1f).

**FIGURE 2:**
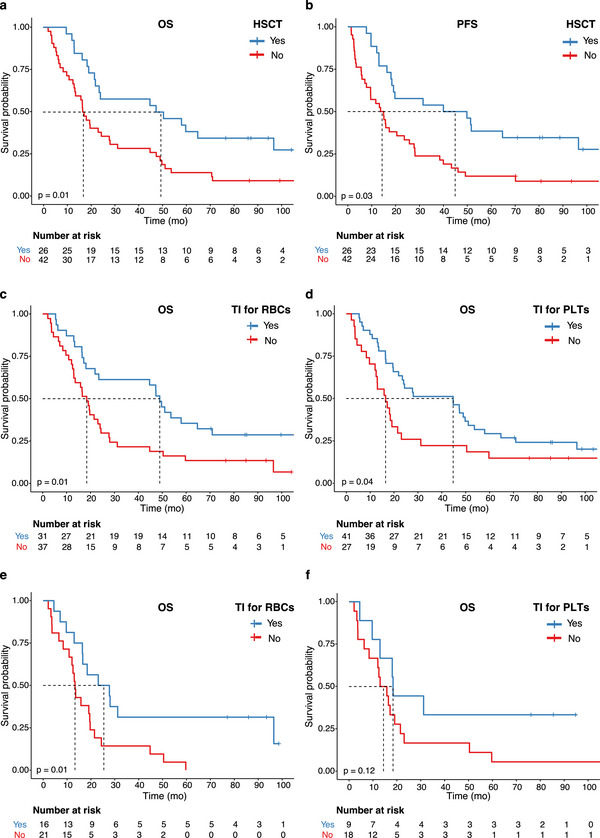
(a) OS comparison between the groups of 5‐AZA‐treated patients who underwent allogeneic haematopoietic stem cell transplantation (HSCT, blue curve) versus the ones without HSCT history (red curve). The mOS of patients undergoing HSCT was 48.8 months (95% CI: 21.9–NA) versus 16.9 months (95% CI: 13.3–28) for the ones with no history of HSCT. (b) PFS comparison between the groups of 5‐AZA‐treated patients who underwent allogeneic HSCT (blue curve) versus the ones without HSCT history (red curve). The mPFS of patients undergoing HSCT was 44.9 months (95% CI: 18.4–NA) versus 14.3 months (95% CI: 9.5–25.8) for the ones with no history of HSCT. (c) OS comparison between the groups of 5‐AZA‐treated patients who were independent on RBC transfusions (i.e. TI for RBCs, blue curve) versus transfusion‐dependent patients (red curve) at 5‐AZA treatment initiation. The mOS of RBC TI patients was 49.0 months (95% CI: 21.9–70.9) and 18.4 months (95% CI: 13.0–24.4) for RBC TD patients. (d) OS comparison between the groups of 5‐AZA‐treated patients who were independent on PLT transfusions (i.e. TI for RBCs, blue curve) versus transfusion‐dependent patients (red curve) at 5‐AZA treatment initiation. The mOS of PLT TI patients was 44.7 months (95% CI: 23.5–53.6) and 16.5 months (95% CI: 12.5–23.1) for PLT TD patients. (e). OS comparison between the groups of RBC transfusion‐dependent patients (*n* = 37) who achieved TI (blue curve) or not (red curve) under 5‐AZA treatment. The mOS of patients achieving RBC TI under 5‐AZA treatment was 25.4 (95% CI: 16.4–NA) vs. 13.3 (95% CI: 10.9–21.5). (f) OS comparison between the groups of RBC transfusion‐dependent patients (*n* = 27) who achieved TI (blue curve) or not (red curve) under 5‐AZA treatment. The mOS of patients achieving PLT TI under 5‐AZA treatment was 18.4 (95% CI: 13.0–NA) versus 14.4 (95% CI: 8.4–23.1) for the group who did not.

HSCT was significantly associated with a better mOS compared with non‐transplanted patients (mOS 48.8 months vs. 16.9 months, *p* = 0.01, Figure 2a). Similarly, mPFS was significantly longer in patients who received HSCT with 44.9 months versus 14.3 months (*p* = 0.002, Figure 2b).

RBC and PLT transfusion dependency at diagnosis were associated with increased mortality with HRs of 1.98 (*p* = 0.01) and 1.7 (*p* = 0.05), respectively (Figure 2c,d), while achieving TI for RBC was associated with a significantly longer mOS of 25.4 months versus 13.3 months (*p* = 0.01) for patients remaining TD (Figure 2e). Although a similar trend was noticed for PLT TI, it did not reach statistical significance (*p* = 0.12, Figure 2f).

To evaluate the relationship between age, sex, TD (RBC and/or PLT) at diagnosis, achieving a CR and undergoing HSCT, with respect to OS, we conducted a Cox multivariate analysis using these covariates. Being RBC TI, undergoing HSCT, and achieving CR appeared to be independent factors associated with better prognosis with HRs of 0.48 (*p* = 0.02), 0.32 (*p* = 0.004) and 0.4 (*p* = 0.02). Age, sex and PLT transfusion‐dependency status at diagnosis did not stand out as independent prognostic factors in light of the other covariates (Table 3).

**TABLE 3:** Multivariate Cox regression analysis.

	Hazard ratios	*p* value
Age	1.0	0.98
Sex (m)	0.94	0.83
RBC TD at 5‐AZA initiation	2	0.02^*^
PLT TD at 5‐AZA initiation	1.7	0.1
CR	0.4	0.02^*^
Allo‐HSCT	0.31	0.004^**^

Abbreviations: 5‐AZA, azacitidine; Allo‐HSCT, allogeneic haematopoietic stem cell transplantation; CR, complete response; PLT, platelets; RBC, red blood cells; TD, transfusion dependence.

* *p* ≤ 0.05.

** *p* ≤ 0.01.

### 3.4 Toxicities and Dose Reductions

Cytopenias were the commonest toxicity, followed by cutaneous reactions. Among cytopenias, Grade 3/4 events were reported in 28 patients (41.1%). Grade 3 or 4 neutropenia was the most frequent (23/68, 33.8%), with thrombocytopenia being second (15/68, 22%) and anaemia third (7/68, 10.3%). Treatment‐related mortality (TRM) related to AZA was considered in non‐previously severely cytopenic patients and when happened before HSCT. TRM was 7.4% with five patients dying from treatment‐related complications (one pneumonia, one duodenal haemorrhage, one septic shock due to pneumonia, one ARDS in bilateral pneumonia and one septic shock due to urinary tract infection).

Dose reductions from the target 100 mg/m^2^ dose were applied in 30 patients (40.5%) due to various reasons, with severe neutropenia/agranulocytosis being the most common (10/30) (Table 4).

**TABLE 4:** Haematologic toxicities and dose reductions.

Dose reduction from 100 mg/m^2^, *N* (%)		Haematologic toxicity Grade 3/4, *N* (%)	
30 (40.5%)		Total	28 (41.1%)
Reasons for dose reduction, *N*		Neutropenia	23 (33.8%)
Fatigue	1	Thrombocytopenia	15 (22%)
Lower dose before HSCT	2	Anaemia	7 (10.3%)
Neutropenia/agranulocytosis	10		
Febrile neutropenia	1		
Thrombocytopenia	6		
Anaemia	1		
Multiple cytopenias/pancytopenia	7		
Renal insufficiency	1		
DLI	1		

Abbreviations: DLI, donor lymphocyte infusion; HSCT, haematopoietic stem cell transplantation.

## 4 Discussion

In this retrospective analysis, we evaluated the efficacy of a higher daily AZA dose regimen with shorter duration in the treatment of MDS (5 days at 100 mg/m^2^/day), resulting in a 500 mg/m^2^ versus 525 mg/m^2^ weekly dose with the standard regimen.

We included a total of 68 patients, 67.6% of whom were men. Male predominance in MDS and CMML is known in the literature, although the men/women ratio in our study is slightly higher than SEER data for MDS (2.09 vs. 1.67) [17]. Similarly, the median age in the cohort is 66 years, which is lower than the median age of diagnosis reported in prospective studies (76 years) [18].

Our reported 62% ORR with approximately 22% of CRs was higher for the MDS with < 10% blast cells when compared to EB‐II and t‐MDS. The median OS of the whole cohort was 22.5 months, with the t‐MDS and MDS‐EB II groups having inferior OS as compared to other MDS. These results are expected, as EB‐II is more prone to AML transformation with inferior survival and it is in consequence classified as an independent disease entity (MDS/AML category) in the recent ICC 2022 classification [19]. Also, t‐MDS, as every secondary therapy‐related myeloid neoplasm, has inferior survival than de novo disease, as confirmed recently in a large population‐based study [20]. Comparison with other real‐life studies using the standard AZA schedule provides more insight evidence to the efficacy of this alternative regimen. Studies in large MDS cohorts with all subgroups [21, 22, 23, 24, 25] reported ORRs between 30% and 55% with CR between 11% and 18%. Median OS were reported to be between 15 and 27 months, but in cohorts that also included low‐risk MDS, which may have accounted for improved outcomes. Our cohort contains only few intermediate or lower‐risk patients (2%, 1/50), meaning that most of the participants had higher‐risk disease (49/50, 98%).

Studies with alternative schedules from the 75 mg/m^2^ dose, most commonly the 5‐2‐2 and 5‐0‐0 schedule, reported mOS between 13 and 19 months with an ORR between 50% and 68% [10, 26, 27]. The 5‐2‐2 schedule seemed to have superior results in some studies with ORR of more than 50% and CR up to 67% [28, 29, 30] and mOS of 19 months [10]. Only few data exist regarding the 100 mg/m^2^ schedule. The retrospective study of Pierdomenico et al. reports an ORR of 48% with CR 16% and TI 62% [31], which are somewhat comparable to this cohort (62%, 22% and 46%, respectively); however, no direct comparison can be made. Higher CR rate in our cohort, compared to most studies on the classical or alternative schedules, cannot be fully explained as cohorts were highly diverse, but maybe higher dose AZA pushes to deeper responses in patients that are sensitive to hypomethylating agents, and thus leading to more CR.

Comparing the primary objectives with the results of the AZA‐001 study [7], we report a similar mOS (24.5 months in AZA‐001 and 22.5 months in our study). CR and PR, however, were higher in our cohort (22% vs. 17% and 40% vs. 12%, respectively), but it should be mentioned that in the AZA‐001, low blast AML were also included, which probably worsens the prognosis of the whole cohort. Lastly, median follow‐up was much longer in this study, with 98 versus 21 months in the AZA‐001, thus underscoring the reliability of our survival results.

Another interesting aspect is the time to best response. In our cohort, the median time was longer for patients who responded to treatment than the time needed to observe refractoriness (CR = 6.9 months, PR = 3.65 months, SD = 2.9 months, PD = 2.6 months). In consequence, better responses occurred progressively over time, while non‐responders could be rapidly identified after two to three cycles. This was corroborated by other AZA studies, which reported a delay of two to three monthly cycles before achieving at least a PR [6, 25, 27, 32]. However, response depth was increased with more cycles administered. For example, van der Helm et al. report an ORR of 49% at three cycles, 74% at six cycles, and 88% after nine cycles [33]. Pierdomenico et al., who also studied 100 mg/m^2^ in 5 days, report four cycles needed to best response [31]. Thus, response to AZA is progressively improving after the first two cycles, with responders reaching their best response after four to six cycles. On the other hand, if the disease is progressing after two cycles, the treatment will probably fail.

HSCT was found to be a beneficial factor for OS and PFS in our cohort, with patients achieving much better results if they could be consolidated with HSCT. This concurs with the existing literature that has proven the survival advantage of HSCT in MDS, whenever feasible [34]. HSCT detains a survival advantage over AZA alone, as proved by Platzbecker et al. [35]. Whether or not AZA bridging should be used before HSCT depends on donor availability, blast count and centre preferences and has been proven to be equal or even superior to intensive chemotherapy before HSCT [36, 37].

As expected, TD for RBC or PLT was associated with an increased mortality and TI during treatment had a significant impact on improving mOS. Treatment‐induced RBC‐TI was reached in 46% of the patients and PLT‐TI in 30% in our cohort, like the AZA‐001, which reported 45% RBC‐TI [7]. Time to TI was 5 months for PLT and RBC, and also demonstrated a significant mOS improvement in AML patients treated with AZA, similar to our results in MDS patients [38].

Concerning toxicities, we report 41% of patients with severe cytopenias (Grade 3 or 4), mostly neutropenias (34%). Almost all prospective AZA trials reported serious cytopenias at higher rates, always surpassing 50% [6, 7, 39]. In real‐life studies such as this one, percentages of reported myelotoxicity were lower, similar to our cohort. Beguin et al. report 29.4% of Grade 3/4 toxicities, while Breccia et al. reported 30% of neutropenias and 25% of thrombocytopenias, similar to our results [22, 30]. Garcia‐Delgado et al. did not find any statistical difference in toxicities between 5‐0‐0, 5‐2‐2 and 7‐0‐0 schedules, with percentages for neutropenia between 20% and 40%, thrombocytopenia between 9% and 15% and anaemia between 7% and 10% [10]. The reason for these differences between prospective and retrospective studies is poorly understood and may be due to exhaustive adverse event report in prospective trials or due to frequent dose adaptations (at the discretion of the treating physician) in the real‐life setting.

ORR for the 17 CMML and MDS/MPN NOS patients was high with 73.6% and 31.5% of CR in the CMML and MDS/MPN NOS groups, respectively. In many studies, CMML cases tend to be analysed together with other MDS, as in the AZA‐001 trial [7], but separate results are rarely reported. In 2011, a retrospective study included 36 CMML patients receiving AZA, with either 75 mg/m^2^ 7 days or 100 mg/m^2^ 5 days. They reported 39% ORR and 11% CR. They also distinguished myelodysplastic CMML (MD‐CMML) from myeloproliferative (MP‐CMML), with the first having better ORR at 55% versus 32% for MP‐CMML [40]. Other studies confirmed the same trend of > 50% ORR in MD‐CMML, whereas leucocytosis > 13 G/L (MP‐CMML) is an adverse response marker [40, 41, 42]. This explains the good results of our CMML cohort, as the median leucocyte count for the included CMML patients was 7.6 G/L (3–75.8). In addition, nearly half of our CMML patients (8/17) were treated with AZA as second‐ or third‐line treatment, often after intensive chemotherapy.

Despite limitations, our data demonstrate that the intensified 100 mg/m^2^ 5‐day schedule is a feasible and safe alternative to the classical 7‐0‐0 regimen. The response rates and the mOS are comparable to the classical AZA‐001 and other retrospective real‐life studies with 75 mg/m^2^ at different formulations.

This shorter schedule is more convenient for patients and more feasible in the ambulatory setting, as outpatient clinics are closed during the weekend.

## Author Contributions

**Gerasimos Tsilimidos**: conceptualization, investigation, methodology, visualization, writing – original draft, writing – review and editing. **Filipe Martins**: formal analysis, visualization, writing – review and editing. **Mariangela Costanza**: writing – review and editing. **Mathilde Gavillet**: conceptualization, writing – review and editing. **Sabine Blum**: conceptualization, methodology, project administration, supervision, writing – review and editing.

## Funding

The authors have nothing to report

## Ethics Statement

This study was approved by the ethics committee of the Canton de Vaud under project number CER‐VD n.2018‐01391.

## Consent

Patients signed the consent form regarding the storage and use of blood and bone marrow samples sent to the central haematology laboratory and/or the general consent form from the University Hospital of Lausanne. For patients who did not sign consent, particularly due to death, this study focuses on retrospective data and has no impact on the patients participating in the study, either in terms of benefit or risk. Their physical and moral integrity are not endangered by this research. Consent in these cases was deemed unnecessary according to national regulations (HRA art.34, HRO art.37.40) and was waived by the local ethics committee (CER‐VD n.2018‐01391).

## Conflicts of Interest

The authors declare no conflicts of interest.

## Data Availability

The data that support the findings of this study are available on request from the corresponding author, GT. The data are not publicly available due to information that could compromise the privacy of research participants.
